# STORMing towards a clear picture of the cytoskeleton in neurons

**DOI:** 10.7554/eLife.06235

**Published:** 2015-02-06

**Authors:** Emerson Stewart, Kang Shen

**Affiliations:** Department of Biology, Stanford University, Stanford, United States; Department of Biology, Stanford University, Stanford, United States, kangshen@stanford.edu

**Keywords:** actin, spectrin, ankyrin, axon, super-resolution, STORM, Mouse, rat

## Abstract

Super-resolution microscopy has shed new light on the formation of the actin-spectrin network in neurons.

**Related research article** Zhong G, He J, Zhuo R, Lorenzo D, Babcock H, Bennett V, and Zhuang X. 2014. Developmental mechanism of the periodic membrane skeleton in axons. *eLife* **3**:e04581. doi: 10.7554/eLife.04581

**Image** The cytoskeleton of neurons contains a network that is made of two proteins (F-actin and spectrin)

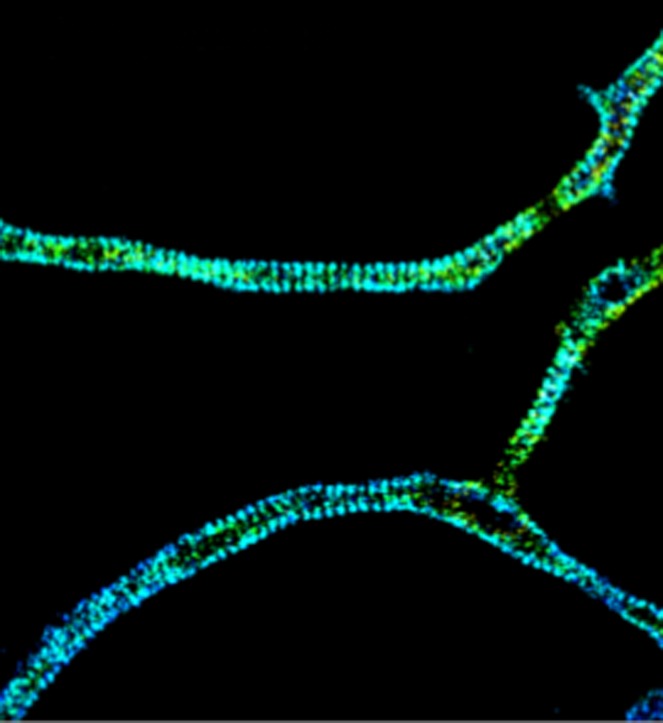

Neurons are highly specialized cells that carry information around the nervous system. Although neurons come in a variety of different shapes and sizes, they share common features: a cell body called the soma that contains the nucleus; projections called dendrites that receive information from other neurons; and projections called axons that pass information to neighboring neurons. A fundamental question in neurobiology is how neurons establish and maintain these distinct features.

Compelling evidence suggests that the cytoskeleton plays a central role in establishing and maintaining the structures of the dendrites and axons that are vital to the various roles of neurons (Susuki and Rasband, 2008; Kapitein and Hoongenraad, 2011; Bennett and Lorenzo, 2013; Machnicka et al., 2014). The small size of the axons and dendrites calls for techniques that offer both high spatial resolution and the ability to distinguish between different molecules (Hirokawa, 1982). Now, in *eLife*, Xiaowei Zhuang of Harvard University and co-workers at Harvard and Duke University—including Guisheng Zhong and Jiang He as joint first authors—provide new insights into how a part of the cytoskeleton called the cortical actin-spectrin network forms in the axons of neurons (Zhong et al., 2014).

The textbook view of this network comes from electron microscopy studies performed on membrane skeletons purified from red blood cells. These studies revealed that the network—which forms around the edge of the cells near the cell membrane—is arranged in a repeating lattice, which is usually hexagonal. Each hexagon consists of short filaments of the protein F-actin at its center and each corner. The F-actin filaments are connected to one another by spectrin, a protein that is usually found in a four-molecule complex made of both α-spectrin and β-spectrin (Figure 1A; Liu et al., 1987).

**Figure 1.**
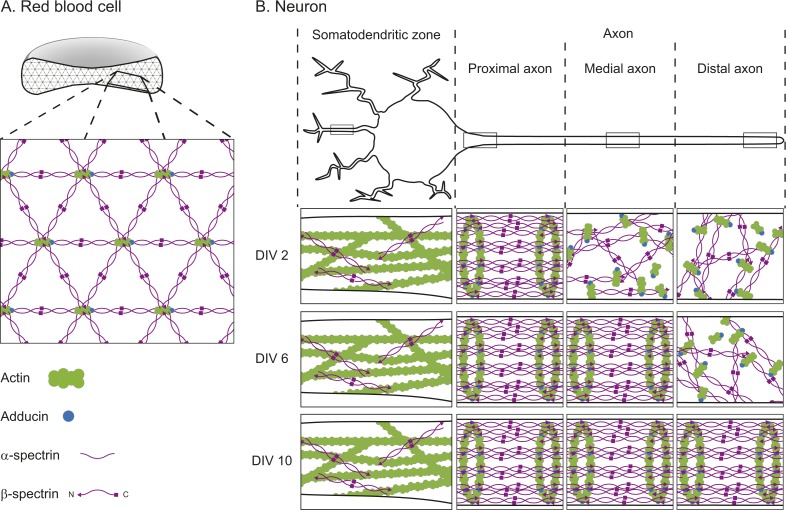
The cortical actin-spectrin networks found in red blood cells and neurons have different structures. (**A**) Schematic diagram of a cross-section through a red blood cell showing the cortical actin-spectrin network. The inset is a detailed view of the network, which is comprised of the proteins F-actin (green), adducin (blue) and spectrin (purple) arranged in a hexagonal lattice structure: short filaments of F-actin capped with adducin are connected by spectrin complexes made of a mixture of α-spectrin and β-spectrin. (**B**) Schematic diagram of a neuron showing the somatodendritic zone (which contains the soma and dendrites; left), and the proximal, medial, and distal sections of the axon. The insets represent detailed views from within the boxed regions for neurons cultured for 2 days in the laboratory (2 DIV, top row), 6 days (6 DIV, middle row), or 10 days (10 DIV, bottom row). At 2 DIV the spectrin rings begin to form in the axon region adjacent to the soma, while F-actin and spectrin in the medial and distal axon remain disorganized. By 6 DIV the spectrin rings have spread to the medial axon, and at 10 DIV the lattice is present throughout the axon. In the dendrites, F-actin is the main contributor to the cortical cytoskeleton, with long filaments that run parallel to the long axis of the dendrite. β-spectrin is present in the dendrites at low concentrations (about half those found in the axon) and it rarely exhibits any periodic structure. DIV: days in vitro.

The primary role of the actin-spectrin network in a red blood cell is to provide mechanical stability and resilience as the cell travels through blood vessels (Bennett and Baines, 2001). Research has suggested a similar role for spectrin in axons, helping them to resist the mechanical stress they experience as an organism moves around (Hammarlund et al., 2007). However, it was unclear whether neurons also have a cortical actin-spectrin network, and if so, whether the architecture is similar to that found in red blood cells.

Zhuang and co-workers recently addressed this question using a super-resolution microscopy technique called stochastic optical reconstruction microscopy (STORM) to examine neurons in cell culture and in brain slices (Xu et al., 2013). In dendrites, long F-actin filaments that run parallel along the length of the dendrites are the primary constituents of the cytoskeleton close to the cell membrane. In contrast, the axon has a cortical actin-spectrin network, but with a different architecture to that of red blood cells. The short F-actin filaments are arranged in rings that wrap around the circumference of the axon. These rings occur in a regular pattern that is repeated along the length of the axon and they are connected by two-molecule complexes containing α-II and β-II-spectrin.

The distinct architecture of this network in axons directs the localization of many proteins including ankyrins and sodium ion channels, underscoring its fundamental importance to the development of neurons. While paradigm shifting, this previous work did not reveal how the network forms in neurons.

Now Zhong, He et al. have used a combination of molecular and genetic approaches, along with STORM, to study how the network forms in neurons grown in cell culture. They began by investigating the relationship between the F-actin and spectrin rings to determine whether the formation of one is dependent on the formation of the other. They found that disrupting either the F-actin structure or the β-II-spectrin was sufficient to prevent the formation of the F-actin and spectrin rings.

Zhong, He et al. also investigated how the network formed over time. They found that, after 2 days, the spectrin rings had begun to form in the section of the axon nearest the soma; by 6 days the periodic structure extended into the middle of the axon, and by 10 days it had reached the other end of the axon (Figure 1B). Although the network started to form near the soma, it only spread in one direction—into the axon. Zhong, He et al. examined the underlying mechanisms that prevented the network forming in the dendrites. They discovered that network formation was tightly correlated to the levels of β-II-spectrin—the level of β-II-spectrin was much higher in the axons than in the dendrites. Also, they found that the protein ankryin B plays an important role in promoting the accumulation of β-II-spectrin within the axon.

The precise details of how ankyrin B works and the mechanisms that determine where the cortical actin-spectrin network starts to develop in the axon remain fertile ground for future studies. Most importantly, the work of Zhong, He et al. demonstrates the potential of super-resolution microscopy for gaining traction on previously intractable problems. As these approaches become more widely used, it will be exciting to see what other areas of biology come into a clear focus.
